# Asarone from Acori Tatarinowii Rhizoma Potentiates the Nerve Growth Factor-Induced Neuronal Differentiation in Cultured PC12 Cells: A Signaling Mediated by Protein Kinase A

**DOI:** 10.1371/journal.pone.0163337

**Published:** 2016-09-29

**Authors:** Kelly Y. C. Lam, Jianping Chen, Candy T. W. Lam, Qiyun Wu, Ping Yao, Tina T. X. Dong, Huangquan Lin, Karl W. K. Tsim

**Affiliations:** 1 Division of Life Science, Center for Chinese Medicine, The Hong Kong University of Science and Technology, Clear Water Bay, Hong Kong, China; 2 HKUST Shenzhen Research Institute, Hi-Tech Park, Nanshan, Shenzhen, Guangdong Province, China; 3 Shenzhen Key Laboratory of Hospital Chinese Medicine Preparation, Shenzhen Traditional Chinese Medicine Hospital, Guangzhou University of Chinese Medicine, Shenzhen, China; University of Texas Health Science Center at Houston, UNITED STATES

## Abstract

Acori Tatarinowii Rhizoma (ATR), the rhizome of *Acorus tatarinowii* Schott, is being used clinically to treat neurological disorders. The volatile oil of ATR is being considered as an active ingredient. Here, α-asarone and β-asarone, accounting about 95% of ATR oil, were evaluated for its function in stimulating neurogenesis. In cultured PC12 cells, application of ATR volatile oil, α-asarone or β-asarone, stimulated the expression of neurofilaments, a bio-marker for neurite outgrowth, in a concentration-dependent manner. The co-treatment of ATR volatile oil, α-asarone or β-asarone, with low concentration of nerve growth factor (NGF) potentiated the NGF-induced neuronal differentiation in cultured PC12 cells. In addition, application of protein kinase A inhibitors, H89 and KT5720, in cultures blocked the ATR-induced neurofilament expression, as well as the phosphorylation of cAMP-responsive element binding protein (CREB). In the potentiation of NGF-induced signaling in cultured PC12 cells, α-asarone and β-asarone showed synergistic effects. These results proposed the neurite-promoting asarone, or ATR volatile oil, could be useful in finding potential drugs for treating various neurodegenerative diseases, in which neurotrophin deficiency is normally involved.

## Introduction

Neurotrophins are groups of growth factors produced naturally in the brain, which promote the growth and survival of neurons. In developing nervous system, the target cells secrete neurotrophins, and therefore neurons received sufficient amount of neurotrophins will survive and vice versa. Abundance of evidence proposing the alteration of neurotrophic factors could be involved in pathophysiology of neurodegenerative disorders [1]. The decreased levels of neurotrophins result in malfunction of development, functional maintenance and survival of neuronal cells. Currently, there are no effective treatment for neurodegenerative diseases, and indeed neurotrophic factor therapy is one of the potential therapeutic approaches [2–3]. However, neurotrophic factors, e.g. nerve grow factor (NGF), does not penetrate the blood brain barrier. Therefore, a neurotrophic factor-stimulating agent that could pass the blood-brain barrier is needed for such therapy.

Acori Tatarinowii Rhizoma (ATR), the rhizome of *Acorus tatarinowii* Schott, is commonly used clinically as a traditional Chinese medicine (TCM) for mental health. ATR is firstly recorded in Divine Husbandman’s Classic of the Materia Medica (206 BC-220 AD), and which has been used for more than 2,000 years. The major active components of ATR are volatile oils, and indeed α-asarone and β-asarone are accounting about 95% of total ATR oil [4–5]. Chinese Pharmacopoeia (2015) specifies that the content of volatile oils shall be not less than 1.0% in ATR. In line to the functions of ATR, the volatile oil showed pharmacological properties of sedative, anti-convulsant, anti-asthmatic, improving intelligence, anti-oxidant, anti-aging and anti-depression [6–8]. Moreover, ATR oil is known to pass the blood brain barrier. In ATR-treated rat, the distribution of oil, e.g. asarone, among the hippocampus, brainstem, cortex and cerebellum did not have significant differences, suggesting its wide effects on the brain [9]. Here, we speculated that ATR volatile oil could possess neurogenesis role in neurons. In cultured PC12 cells, the neurotrophic properties of ATR volatile oil, α-asarone and β-asarone in inducing neurite outgrowth were determined. In addition, the possible role of ATR oil in a protein kinase A (PKA)-cAMP responsive element binding protein (CREB) signaling during the induction of neuronal differentiation was revealed.

## Materials and Methods

### Chemicals and Plant Materials

α-Asarone (>98%) and β-asarone (>98%) were kindly provided by Testing Laboratory for Chinese Medicine (Hong Kong, China). Ultra-pure water was prepared from a Milli-Q purification system (Millipore, Molsheim, France). ATR herbs were purchased from herbal market in Hong Kong and mainland China. These materials were authenticated by Dr. Tina T. X. Dong, according to their morphological characteristics, which matched the descriptions stipulated in Chinese Pharmacopoeia (2015). The voucher specimens (ATR-1-2014) were deposited in Center for Chinese Medicine R&D at The Hong Kong University of Science and Technology. The total volatile oil from ATR was obtained by volatile oil extraction method, as stated in Chinese Pharmacopoeia (2015). Fifty grams of ATR herb was minced and soaked in water in the proportion of 1:8 (w/v) overnight. The mixture was submitted to hydro-distillation in a Clevenger-type apparatus for 4 hours. The ATR volatile oil was dried over anhydrous sodium sulfate. The yield of volatile oil was 1 mL (2%, v/w), and the volatile oil sample was stored at -20°C until analyze.

### PC12 Culture

Pheochromocytoma PC12 cells, derived from rat adrenal medulla, were obtained from American Type Culture Collection (Manassas, VA) and cultured in Dulbecco’s modified Eagle’s medium (DMEM), supplemented with 6% fetal bovine serum and horse serum, 100 units/mL penicillin and 100 μg/mL streptomycin in a humidified CO_2_ (7.5%) incubator at 37°C. Fresh medium was applied every other day. Culture reagents were from Invitrogen (Carlsbad, CA). Cell viability was assessed by MTT [3-(4, 5-dimethyl-2-thiazolyl)-2, 5-diphenyl-2H-tetrazolium bromide] assay. Cells were plated in 96-well plate for 24 hours and treated with drugs for 48 hours before adding MTT. Then the cells were incubated with MTT for another 3 hours at 37°C. After that, absorbance of 570 nm was measured in a microplate reader (Thermo Fisher Scientific, Waltham, MA) [10].

### Transfection of DNA Constructs

To study the effects of α-asarone, β-asarone, or ATR volatile oil on the expression of neurofilaments, two DNA constructs of pNF68-Luc and pNF200-Luc reporter genes in the pLightSwitch_Prom vector were purchased from Switchgear Genomics (Menlo Park, CA). For the transcriptional activation of cAMP-dependent pathway, three repeats of cAMP response elements (CRE: 5’-TGA CGT CA-3’) were sub-cloned into pTAL-Luc, a promoter-reporter vector (Clontech, Mountain View, CA) that having a downstream reporter of firefly luciferase gene; this DNA construct was named as pCRE-Luc. Cultured PC12 cells were transiently transfected with pNF68/pNF200-Luc or pCRE-Luc by lipofectamine 3000 reagent (Invitrogen). For each 24-well plate, 12 μL of lipofectamine 3000 was added into 600 μL opti-medium and incubated for 5 minutes. Then 600 μL opti-medium containing 6 μg DNA was added into 600 μL of lipofectamine 3000 solution. After incubation of 20 minutes, 50 μL of the mixture was applied to each well. Fresh medium was changed after 16 hours. The transfection efficiency was about 40%, which was determined by another control plasmid having β-galactosidase, under a cytomegalovirus enhancer promoter. Luciferase activity was assay by a luciferase assay kit (Tropix Inc., Bedford, MA). Briefly, the cell lysate (50 μL) was transferred to the 96-well assay plate and set on the luminance reading machine (Promega, Madison, WI). After luciferase reagent A (25 μL) and luciferase reagent B (100 μL) were added into each well automatically, the luciferase activity was assessed by the performance of bioluminescent reading. The readings of bioluminescent intensity were then normalized by amount of protein for each sample [11].

### Neurite Outgrowth Assay

Cultured PC12 cells were treated with α-asarone, β-asarone, or ATR volatile oil with/without low concentration NGF (0.5 ng /mL) for 48 hours; fresh medium and reagents were supplied every 24 hours. A light microscope (Zeiss Group, Jena, Germany) equipped with a phase-contrast condenser, 10x objective lens and a digital camera (Zeiss Group) were used to capture the images with the manual setting. For analyzing the number and length of neurite, approximately 100 cells were counted from at least 10 randomly chosen visual fields for each culture. Using the SPOT basic software (Diagnostic Instruments, MI), the cells were then analyzed for number and length of neurite. The cells were scored as differentiated if one or more neurites was longer than diameter of cell body, and they were also classified to different groups according to the length of neurite that it possessed, i.e. <15 μm, 15–30 μm and >30 μm [12].

### Polyacrylamide Gel Electrophoresis

For the study on the expression of neurofilament, PC12 cells (8 × 10^4^ cells/mL) were seeded into 12-well plates in normal serum medium for 24 hours and then transferred to low serum (1% FBS, 1% HS, 100 U/mL penicillin, and 100 μg /mL streptomycin) as indicated for 3 hours prior to exposure of the asarone or ATR volatile oil in the absence or presence of H89 or KT5720 (Sigma, St. Louis, MO). After 48 hours of treatment, the cultures were collected in high salt lysis buffer (1 M NaCl, 10 mM HEPES, pH 7.5, 1mM EDTA, 1 mM EGTA, 0.5% Triton X-100), followed by centrifugation at 16,100 g for 10 minutes at 4°C. Total protein content was measured by using Bradford’s method with a kit from Bio-Rad Laboratories (Hercules, CA). Samples of equal amounts of total protein were treated with 2X direct lysis buffer (0.125 M HCl, pH 6.8, 4% SDS, 20% glycerol, 2% 2-mercaptoethanol, and 0.02% bromophenol blue) and boiled for 10 minutes before an 8% gel electrophoresis was performed.

### Western Blot and Phosphorylation Study

PC12 cells were seeded onto a 12-well plate. After the degree of confluence reached to >90%, the culture medium was changed to DMEM without serum over 3 hours. The cells were treated with drug at different time points (0, 5, 10, and 30 minutes) in the absence or presence of low concentration of NGF (0.5 ng/mL). Then, the cells were harvested, digested with 200 μL of 2x direct lysis buffer, and boiled for 10 minutes before an 8% gel electrophoresis was performed. Following the electrophoresis, the proteins were transferred to the nitrocellulose. Transfer and equal loading of samples were confirmed by staining with Ponceau-S. The nitrocellulose was blocked with 5% fat-free milk in Tris-buffer saline/0.1% Tween 20 (TBS-T), and then incubated in the primary antibodies, diluted in 2.5% fat-free milk in TBS-T, over night at 4°C. The primary antibodies used were: anti-NF200 (1:1,000, Sigma), anti-NF160 (1:2500, Sigma), anti-NF68 (1:2,500, Sigma), anti-GAPDH (1:10,000, Abcam Ltd. Cambridge, UK), anti-phospho-CREB (1:2,500, Cell Signaling, Danvers, MA), anti-CREB (1:2,500, Cell Signaling). After that, the nitrocellulose was rinsed with TBS-T and incubated for 2 hours at the room temperature in peroxidase (HRP)-conjugated anti-mouse secondary antibody (Invitrogen), or peroxidase (HRP)-conjugated anti-rabbit secondary antibody (Invitrogen), diluted in 2.5% fat-free milk in TBS-T. After intensive washing with TBS-T, the immune complexes were visualized using the enhanced chemi-luminescence (ECL) method (GE Healthcare, Piscataway, NJ). The intensities of the bands in the control and different samples, run on the same gel and under strictly standardized ECL conditions, were compared on an image analyzer, using a calibration plot constructed from a parallel gel with serial dilutions of one of the sample.

### Calculation of Drug-To-Drug Synergism

The drug interaction was examined by using the multiple drug analysis according to the median-effect principle described by Chou [13–14]. The dose effective curves for each drug and two drug mixture in different concentrations were employed by the median effect equation (*F*_*a*_*/F*_*u*_ = (*D/D*_*m*_)^m^) where *D* is dose; *D*_*m*_ is the dose required for 50% effect; *F*_*a*_ is the fraction effected by *D*; *F*_*u*_ is the unaffected fraction (1-*F*_*a*_); and m is the coefficient of sigmoidicity of dose-effect curve. A combination index (CI) was determined by the classical isobologram equation of Chou-Talalay: CI = (*D*)_1_/(*Dx*)_1_+(*D*)_2_/(*Dx*)_2_, was the dose of drug 1 required to produce x% effect alone; (*Dx*)_1_ was the dose of drug 1 required to produce the same x% effect in combination with *(D)*_2_; *(Dx)*_2_ was the dose of drug 2 required to produce x% effect alone; and *(D)*_2_ was the dose of drug 1 required to produce the same x% effect in combination with *(D)*_1_. CI values referred to CI close to 1 = additive effect; CI >1 = antagonistic effect; and CI <1 = synergistic effect. For dose reduction index (DRI), it indicated how much the dose of each drug in a synergistic combination could be reduced at a given effect level (i.e., at x% inhibition) compared with the doses of each drug alone. The DRI value was calculated as (DRI)_1_ = (*Dx*)_1_/(D)_1_ and (DRI)_2_ = (*Dx*)_2_/(D)_2_.

### Statistical Analysis

Data were expressed as the mean ± standard error of the mean (SEM) for *n* = 3–5. Statistical tests were performed by one-way ANOVA. In the statistical analyses, differences were classed as significant (*) for values of *P* < 0.05, more significant (**) for values of *P* < 0.01, and highly significant (***) for values of *P* < 0.001.

## Results

### ATR Volatile Oil, α-Asarone, or β-Asarone Induces the Transcriptional Activation of Neurofilament Promoters

**Fig 1:**
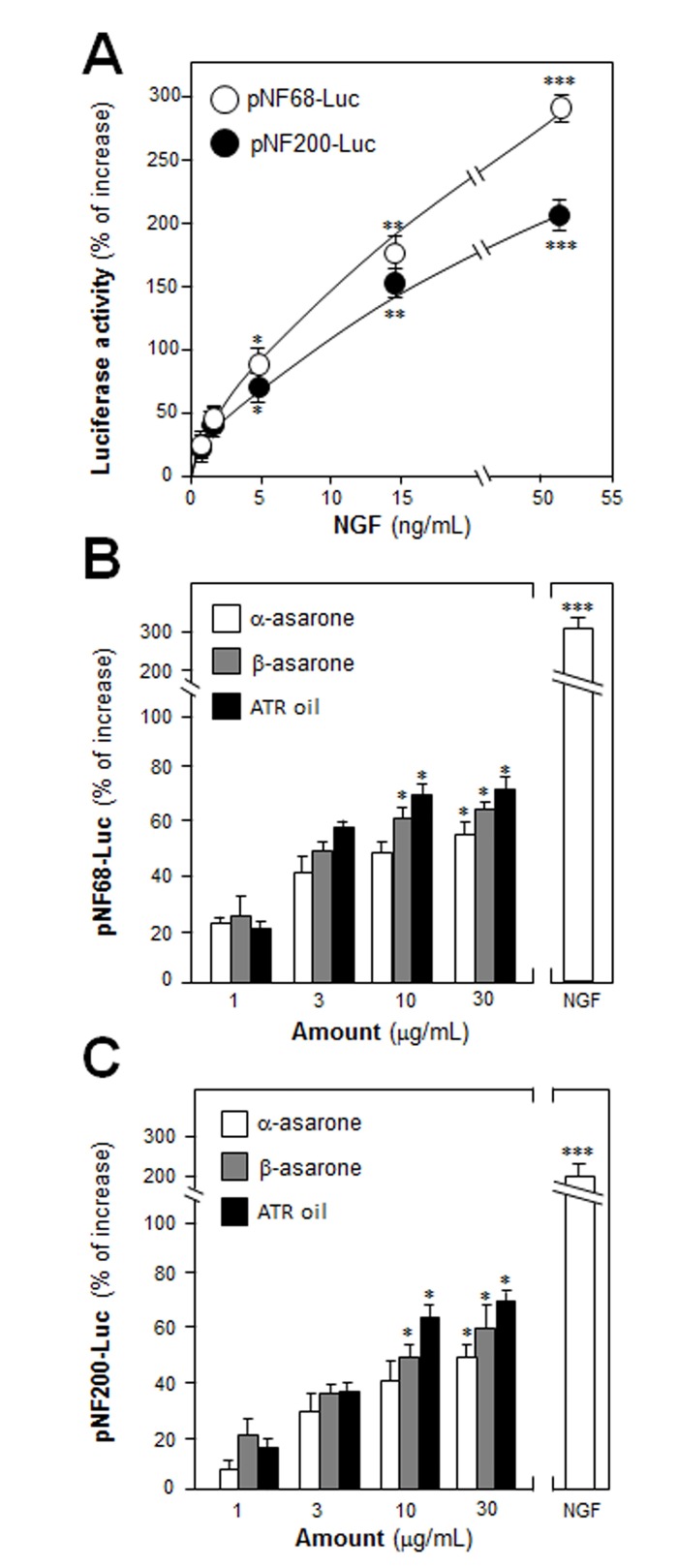
ATR volatile oil, α-asarone or β-asarone, induces the transcriptional activation of neurofilament promoters in cultured PC12 cells. (A) PC12 cells were transfected with pNF68/pNF200-Luc promoter and treated with series dose of NGF for 48 hours. (B & C) ATR volatile oil, α-asarone or β-asarone, was applied onto the cells after transfected with pNF68/200-Luc, as in (A) for 48 hours. The cell lysates were collected to determine the luciferase activity. NGF at 50 ng/ mL served as a control. Values are Means ± SEM, *n* = 3, each with triplicate samples.

PC12 cell stops dividing and terminally differentiated when treated with NGF: this is an excellent model in analyzing the consequences during neuron differentiation. ATR volatile oil was chemically standardized (S1 Fig), and both asarone were over 98% purity. The relative amount of α-asarone and β-asarone in ATR volatile oil was 17% and 76%, respectively. ATR volatile oil, α-asarone or β-asarone, was applied onto cultured PC12 cells in a concentration up to 30 μg/mL: this concentration showed no effect on cell number (S2 Fig). PC12 cells were stably transfected with neurofilament promoter-reporter constructs tagged with luciferase reporter gene (i.e. pNF68-Luc and pNF-200-Luc). NGF, a positive control, induced the promoter activities in dose-dependent manners (Fig 1A). The pNF68/200-Luc stably transfected PC12 cells were treated with ATR volatile oil, α-asarone or β-asarone, for 48 hours: the treatment induced both NF68 and NF200 promoter activities in dose-dependent manners, and the maximal induction of pNF68/200-Luc transcriptional activity was over 70% increase (Fig 1B & 1C).

### ATR Volatile Oil, α-Asarone or β-Asarone, Potentiates NGF-Induced Neurite Outgrowth and Neurofilament Expression

**Fig 2:**
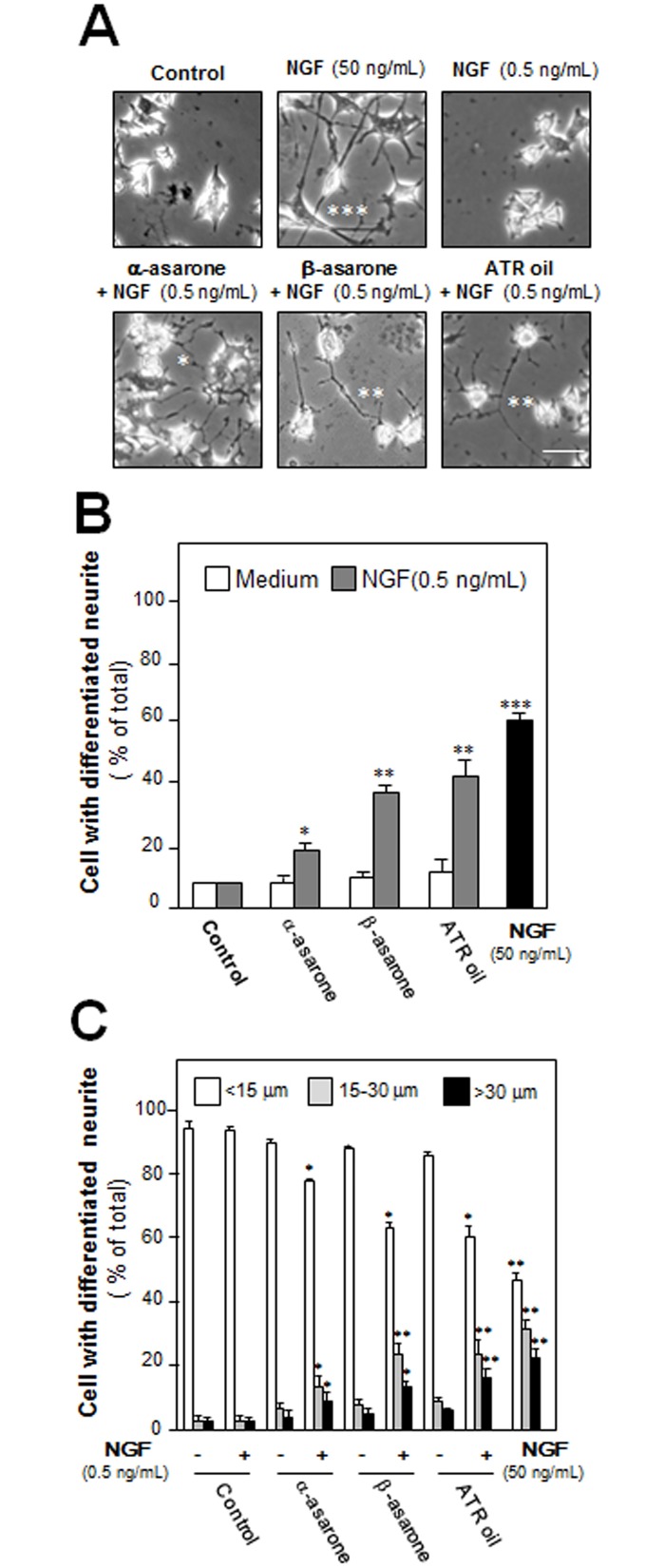
ATR volatile oil, α-asarone or β-asarone, potentiates NGF-induced neurite outgrowth in PC12 cell. (A) Cultures were co-treated with ATR volatile oil, α-asarone or β-asarone, at 30 μg/mL with NGF (0.5 ng/mL) for 48 hours. NGF at 50 ng/mL served as a control. Then, the cells were fixed with ice-cold 4% PFA. Scale bar = 10 μm. Representative images were shown. The percentage of differentiated cells (B) and length of neurite (C) were counted as described in Method section. Values were expressed as % of cells in 100 counted cells, Mean ± SEM, *n* = 4. Each with triplicate samples. * p < 0.05; ** p < 0.01; *** p < 0.001 as compared to the control group.

**Fig 3:**
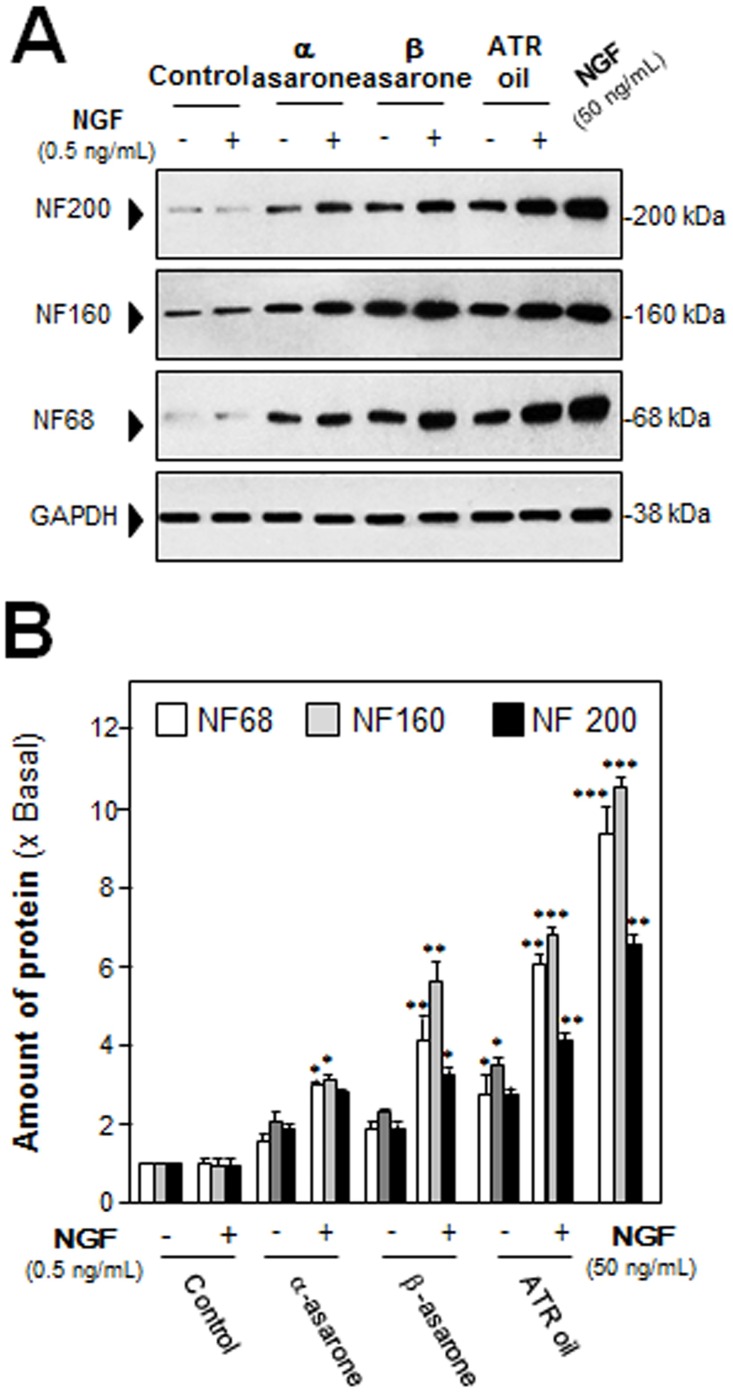
ATR volatile oil, α-asarone or β-asarone, potentiates NGF-induced expression of neurofilaments. (A) Cultures were co-treated with ATR volatile oil, α-asarone or β-asarone, at 30 μg/mL with NGF (0.5 ng/mL) for 48 hours. NGF at 50 ng/mL served as a control. The cell lysates were collected to determine the expression of NF68, NF160 and NF200. GAPDH served as loading control. (B) Quantification from the blots by a densitometer was shown. Values were expressed as the fold of change (x Basal) against the control (no treatment; set as 1), and in Mean ± SEM, *n* = 4, each with triplicate samples.

**Fig 4:**
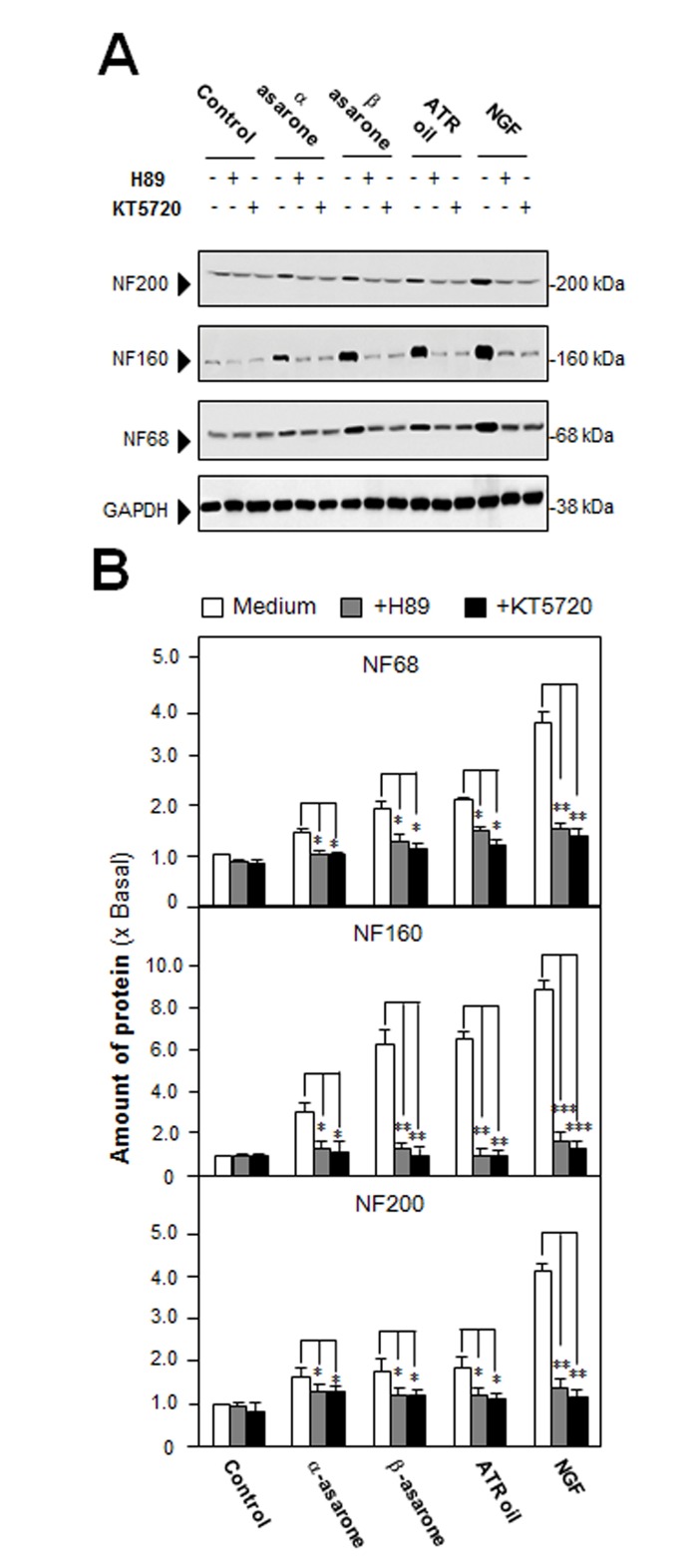
Inhibition of PKA suppresses neurofilament expression, induced by ATR volatile oil, α-asarone or β-asarone, in cultured PC12 cells. (A) Cultured PC12 cells were pre-treated with or without PKA inhibitor, H89 (5 μM) or KT5720 (1 μM), for 3 hours, and then treated with ATR volatile oil, α-asarone, β-asarone, at 30 μg/ mL, or NGF at 50 ng/mL, for 48 hours. The cell lysates were collected to determine the expressions of NF68, NF160, and NF200. GAPDH served as a loading control. (B) Quantification from the blots by a densitometer was shown. Values were expressed as the fold of change (x Basal) against the control (no treatment; set as 1), and in Mean ± SEM, *n* = 4, each with triplicate samples. * p < 0.05; ** p < 0.01; *** p < 0.001.

After the treatment of NGF (50 ng/mL) in cultured PC 12 cells, the morphological change was observed, i.e. longer neurites were protruded from the cell bodies (Fig 2A). This NGF treatment resulted in more than 60% conversion of differentiated cells containing significant extension of neurites (Fig 2B). To evaluate the efficacies of ATR volatile oil, α-asarone or β-asarone, in PC12 differentiation, the treated cells were counted for neurite outgrowth, which showed an increase of differentiated cell by ~15%, but not significant (Fig 2B). In parallel, the ATR oil-induced neurofilament expression was also determined. After the treatment of ATR volatile oil, α-asarone or β-asarone, for 48 hours in cultured PC 12 cells, the expressions of NF68, NF160 and NF200 were markedly increased (Fig 3A & 3B). By treatment of 30 μg/mL α-asarone, or β-asarone, or ATR volatile oil, the expressions of NF68 and NF200 were increased by ~ 2-fold; while NF160 was increased by ~3-fold. Since asarones or ATR volatile oil did not seem to have a significant effect on neurite outgrowth of PC12 cells, we aimed to determine the potentiation effects of ATR volatile oil, α-asarone or β-asarone, together with low dose of NGF. NGF at 0.5 ng/mL failed to induce the neurite extension, which was chosen for the co-treatment analyses (Figs 1A & 2A). The co-treatment of ATR volatile oil, α-asarone or β-asarone, with low dose of NGF significantly potentiated the number of differentiated cell, as well as the length of neurite, from ~20% to 40% (Fig 2B & 2C). The induction of neurofilament was also revealed in the co-treatment of ATR volatile oil, α-asarone or β-asarone, with low dose of NGF: the co-treatment robustly increased the expression of neurofilaments, i.e. NF68, NF160 and NF200. The induction of neurofilament was higher than the induction under single treatment of ATR volatile oil, α-asarone or β-asarone (Fig 3A & 3B). Furthermore, ATR without volatile oil could not induce the transcriptional activation of neurofilament promoters and neurofilament expression in cultured PC12 cells (S3 Fig). To explore the possible mechanism of the ATR oil-induced neuronal differentiation, PC12 cells were pre-treated with PKA specific kinase inhibitor H89, or KT5720, for 3 hours before the treatment of ATR volatile oil, α-asarone or β-asarone. The inhibitors blocked significantly the induction of neurofilaments (Fig 4A & 4B). As a control, the inhibitors also blocked the NGF-induced neurofilament expression.

### ATR Volatile Oil, α-Asarone or β-Asarone, Induces Phosphorylation of CREB

**Fig 5:**
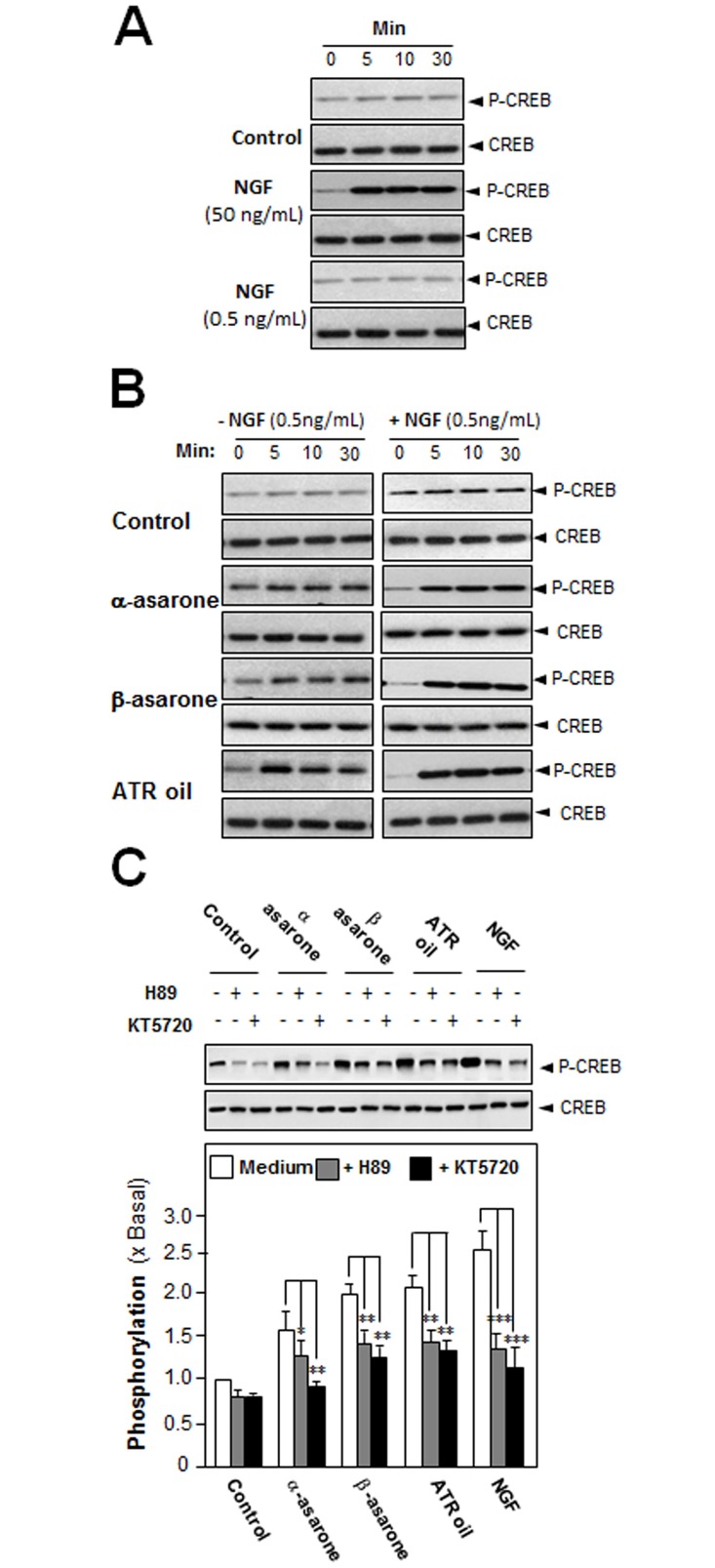
ATR volatile oil, α-asarone or β-asarone, induces phosphorylation of CREB in cultured PC12 cells. (A) Cultured PC12 cells, serum starved for 5 hours, were treated with NGF at high concentration at 50 ng/mL, or low concentration at 0.5 ng/mL. (B) ATR volatile oil, α-asarone or β-asarone, at 30 μg/mL, with or without NGF at 0.5 ng /mL, for different time. (C) Cultured PC12 cells, serum starved for over 5 hours, were pre-treated with or without PKA inhibitor, H89 (5 μM) or KT5720 (1 μM), for 3 hours prior to the treatment with NGF (50 ng/mL), or ATR volatile oil, or α-asarone, or β-asarone, for 10 minutes. Total CREB and phosphorylated CREB (~42 kDa) were revealed by using specific antibodies (upper panel). Quantification plot is shown in histograms (lower panel). Data are expressed as the fold of change (x Basal) against the control (no treatment; set as 1), Mean ± SEM, *n* = 5, each with triplicate samples. Statistical comparison was made with the H89-treated or KT5720-treated group; * p < 0.05; ** p < 0.01; *** p < 0.001.

An increase in intracellular level of cAMP has been shown to induce neuronal differentiation and to co-operate with NGF in inducing PC12 cell neurite outgrowth in a Ras-dependent manner [15]. In the cAMP signaling, PKA has a role in the transcriptional control of neurofilament genes, as well as in neuron differentiation [16]. In order to find out the potency of ATR volatile oil, α-asarone or β-asarone, in stimulating neurite outgrowth and neurofilament expression via the cAMP pathway, the phosphorylation of CREB (cAMP-responsive element binding protein) was evaluated. NGF at 50 ng/mL induced the phosphorylation of CREB robustly, serving as a positive control. Low concentration of NGF (0.5 ng/mL), a concentration used for co-treatment, did not induce phosphorylation of CREB (Fig 5A). ATR volatile oil, α-asarone or β-asarone, was applied onto the serum-starved PC12 cultures, which induced CREB phosphorylation (~43 kDa), significantly (Fig 5B **left panel**). The co-treatment with NGF at low concentration markedly induced the phosphorylation, which was peaked at 5 minutes, and then gradually decreased after 10 minutes (Fig 5B **right panel**). The CREB phosphorylation, induced by ATR volatile oil, α-asarone or β-asarone, could be partially blocked by H89 or KT5720, inhibitors of PKA (Fig 5C).

### ATR Volatile Oil, α-Asarone or β-Asarone, Induces cAMP-Mediated Transcriptional Activity

**Fig 6:**
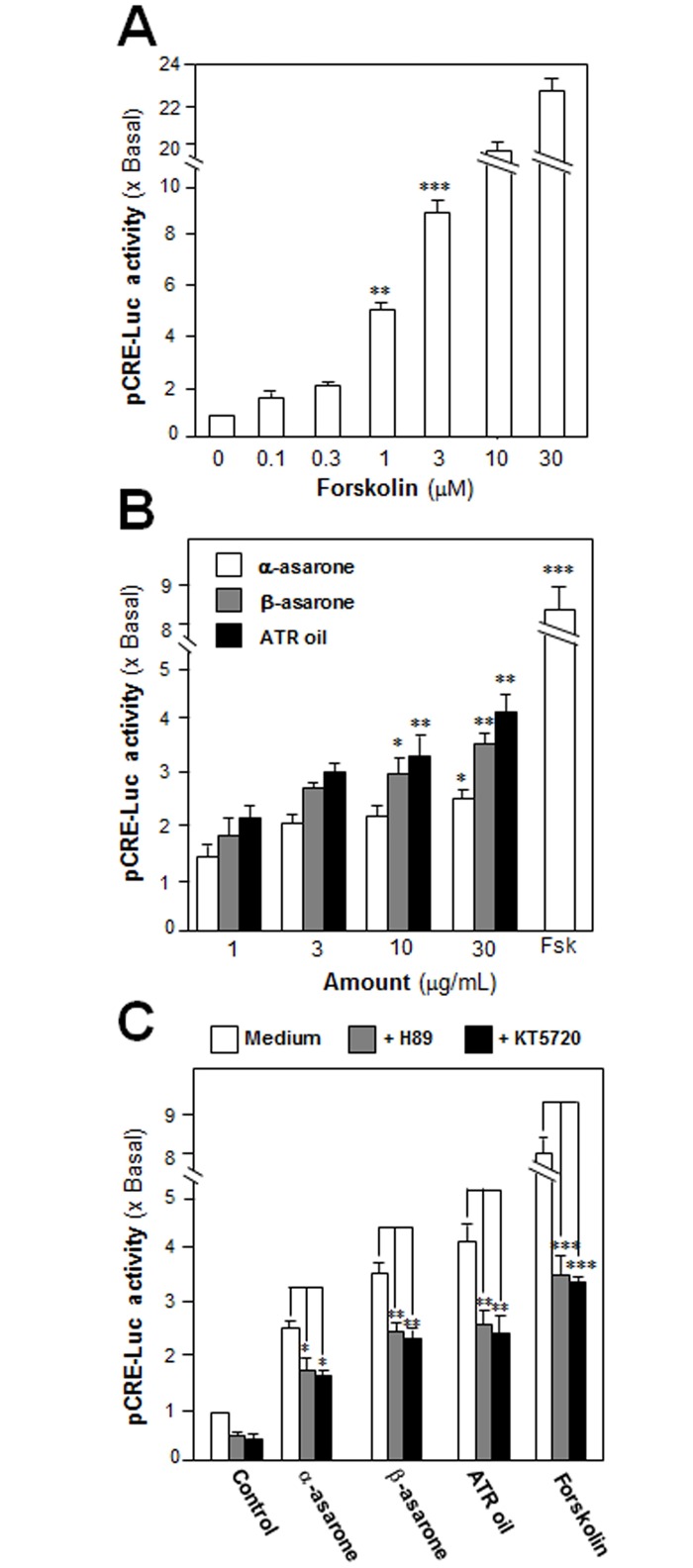
ATR volatile oil, α-asarone or β-asarone, induces cAMP-mediated transcriptional activity in cultured PC12 cells. (A) Cultured PC12 cells, transfected with pCRE-Luc, were treated with series concentration of forskolin for 48 hours. (B) The pCRE-Luc transfected PC12 cells were treated with series concentration of ATR volatile oil, α-asarone or β-asarone, for 48 hours. (C) The pCRE-Luc transfected PC12 cells were pre-treated with PKA inhibitor, H89 (5 μM) or KT5720 (1 μM), for 3 hours, and then treated with ATR volatile oil, α-asarone or β-asarone, at 30μg/mL, or forskolin (3 μM), for 48 hours. The cell lysates subjected to luciferase assay. Values were expressed as the fold of increase to basal reading (DMSO-treated culture as control), and in Mean ± SEM, where *n* = 4, each with triplicate samples. * p < 0.05; ** p < 0.01; *** p < 0.001 as compared to the control group.

An activation of cAMP pathway is essential for neuronal differentiation of PC12 cells [17]. Increase of cAMP concentration by activation of adenylate cyclase leads to activation of PKA, which travels into nucleus and phosphorylated CREB. Activation of CRE transcribes a series of genes related to neuronal differentiation. To investigate the transcriptional activation of CRE, a luciferase-reporter construct (pCRE-Luc), containing three copies of CRE, derived from the promoter, and tagged upstream of a luciferase gene, was transfected into PC12 cells to evaluate the transcriptional activity of ATR volatile oil, α-asarone or β-asarone. The adenylate cyclase activator, forskolin, has been shown to stimulate adenylate cyclase in PC12 cells, as such to raise intracellular cAMP level [18]. Forskolin served as a control here to activate pCRE-Luc at different concentrations (Fig 6A). In pCRE-Luc-transfected PC12 cells, ATR volatile oil, α-asarone or β-asarone, induced the luciferase activity in a dose-dependent manner (Fig 6B). To further confirm the possible mechanism of ATR volatile oil, α-asarone or β-asarone, in cAMP-dependent pathway, the pCRE-Luc transfected PC12 cells were pre-treated with H89 or KT5720 for 3 hours. After the treatment, the transcriptional activity of pCRE-Luc was markedly decreased (Fig 6C). This result indicated a possible relation between the function of α-asarones, β-asarone or ATR volatile oil, in a cAMP-dependent pathway, which could be a cause for induction of neurite outgrowth of PC12 cells.

### α-Asarone and β-Asarone Synergistically Increase Transcriptional Activation of Neurofilament Promoters

**Fig 7:**
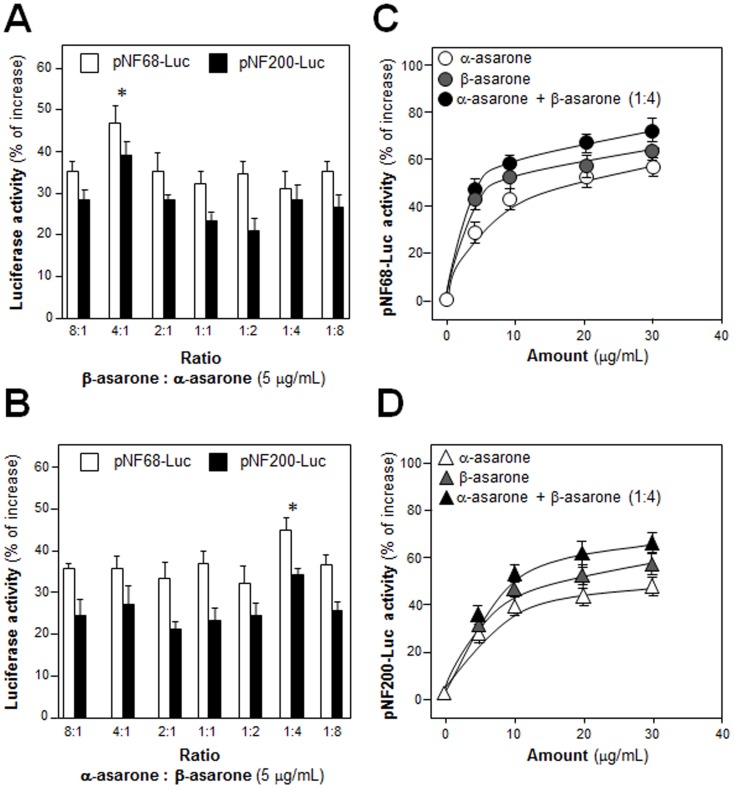
Different combination ratios of α-asarone and β-asarone on increasing NF promoter activity. (A) The concentration of α-asarone of set at 5 μg/mL, and the concentration of β-asarone was varied (0.625–40 μg/mL) to form different ratios from 8:1 to 1:8. A mixture of asarone in defined ratio/concentration was applied onto pNF68/200-Luc transfected PC12 cells. The luciferase activity was determined after 48 hours. (B) The concentration of β-asarone of set at 5 μg/mL, and the concentration of α-asarone was varied (0.625–40 μg/mL) to form different ratios from 8:1 to 1:8. The cell treatment was as in (A). (C) Combination of α-asarone and β-asarone in 1:4 ratio, α-asarone or β-asarone were applied onto pNF68-Luc transfected PC12 cells in different concentrations. The luciferase activity of each sample was determined after 48 hours. (D) The treatment was as in (C) onto pNF200-Luc transfected PC12 cells. Values are expressed as percentage of increase as compared to control (without drug treatment), and in Mean ± SEM, *n* = 4, each with triplicate samples. * p < 0.05; ** p < 0.01; *** p < 0.001.

In the cell assays, ATR volatile oil was more active than α-asarone or β-asarone. A possible synergy within ATR volatile oil could be mediated by α-asarone together with β-asarone. In order to figure out this hypothesis, the synergistic effect of α-asarone with β-asarone in the promoter activity assay was tested. α-Asarone and β-asarone were mixed together by different ratios from 8:1 to 1:8 of α-asarone: β-asarone before application to the culture. The ratio 1:4 of α-asarone: β-asarone was identified as the best ratio (Fig 7A & 7B). To support the nomination of this best ratio, combination of α-asarone: β-asarone in 1:4 was then compared with α-asarone or β-asarone alone. In the activation of pNF68-Luc, the combination of asarone showed significant higher effect than that of α-asarone or β-asarone alone, both in terms of effective dose and maximal activation (Fig 7C). The activation of combination of asarone was similar in the case of pNF200-Luc (Fig 7D), i.e. better than that of α-asarone or β-asarone alone.

**Fig 8:**
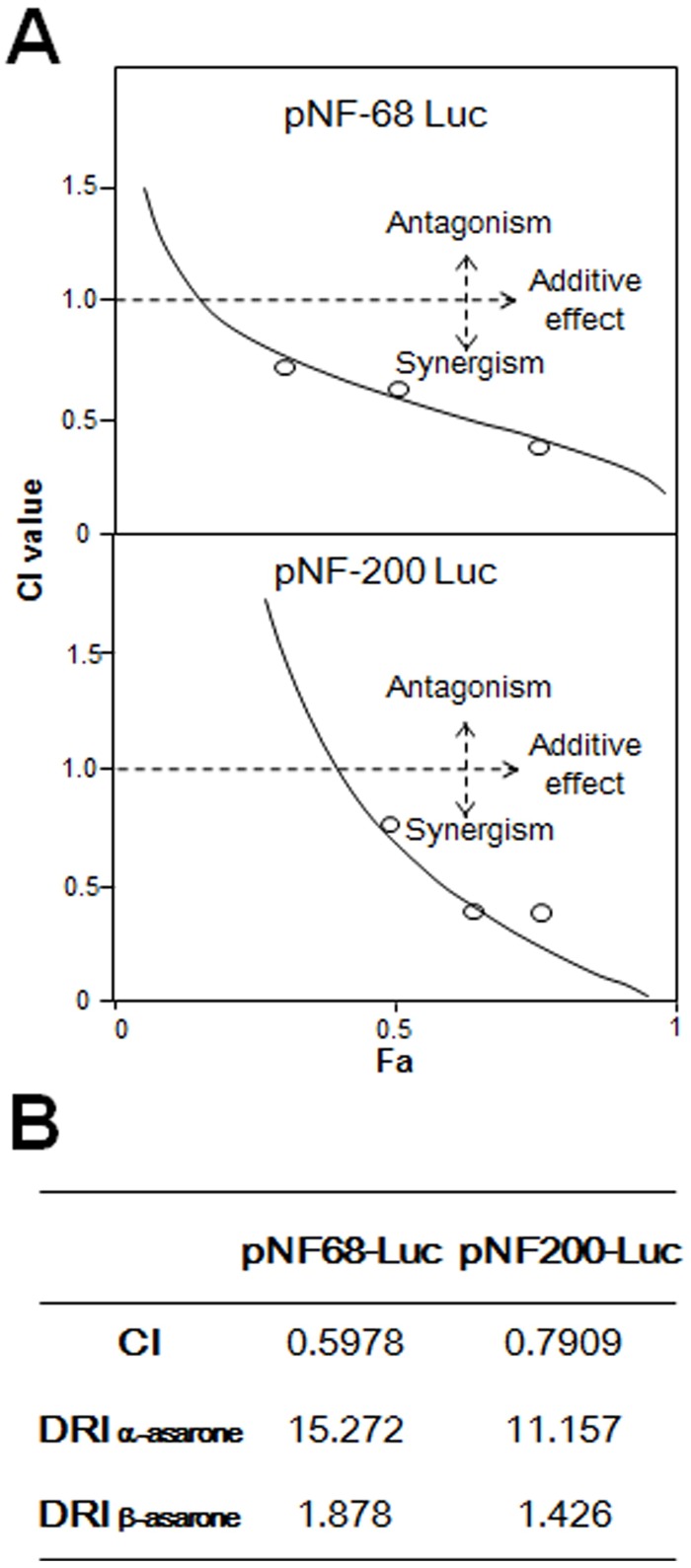
Synergism of α-asarone and β-asarone by median-effect principle. (A) Data from Fig 7 was analyzed by Chou-Talalay method, as described in the section of Materials and Methods. The plots of Fa-CI were demonstrated. The line of co-treatment of α-asarone and β-asarone was in the section of synergism. (B) Data were used to generate the CI and two DRI (for α-asarone and β-asarone) values.

The synergistic effect of α-asarone: β-asarone in 1: 4 ratio was further tested using median-effect equation (Fig 8A). According to the classical isobologram equation of Chou-Talalay: CI = (*D*)_1_/(*Dx*)_1_+(*D*)_2_/(*Dx*)_2_, the calculated CI values from the pairing of the two asarone in both promoter were smaller than 1 (Fig 8B). These results therefore clearly indicated that the combination of α-asarone and β-asarone could produce synergistic effect in stimulating the promoter activities in PC 12 cells. In parallel, the DRI values for α-asarone and β-asarone were range from 1.4–15.3 (Fig 8B), which are greater than 1, suggesting that a dose reduction might be applied during the therapeutic applications.

## Discussion

Neurofilaments are the major cytoskeletal element in neurite, which accumulate in many neurological diseases, e.g. Charcot-Marie-Tooth disease, Parkinson’s disease and amyotrophic lateral sclerosis [19]. The expression levels of two prominent neurofilaments, NF68 and NF200, could serve as markers in early and late stages of neuronal differentiation, respectively. NGF is one of the key modulators of neurite outgrowth during development, and indeed many neurodegenerative diseases are associated with NGF insufficiency, e.g. depression and Alzheimer’s disease [20–21]. NGF achieves its function by binding and activating TrkA receptor on neuronal cells. The NGF-activated TrkA stimulates downstream signaling pathways, which results in neuronal differentiation and promoting cell survival [22]. Here, we showed that ATR oil, in particular asarone, could potentiate a low level of NGF in stimulating neurite outgrowth in cultured PC12 cells. Thus, the intake of ATR could improve those neurodegenerative diseases suffering from a deficiency of NGF in the brain; however, this notion has to be verified by animal study.

ATR is a well-known TCM for treating central nervous system (CNS)-related disorders [23–24]. This herb is reported to be responsible for various CNS pharmacological disorders, such as epilepsy, cerebrovascular diseases and Alzheimer’s disease [25–27]. In addition, the extracts of herbal decoction, containing ATR as a main component, are clinically used for improvement of learning, stroke and anti-depression [28–30]. Neurotrophic factors promotes neurogenesis and maturation of progenitor cells by enhancing GABA release in the subgranular zone [31]. Neurogenesis is vital for spatial learning, as well as emotion state control in both rodents and primates [32–33]. Moreover, stimulation of the neurogenesis speeds up the therapeutic process and leads to fuller compensation of cognitive function [34]. Our study was in good agreement with a previous report that asarone could facilitate the proliferation of PC12 cells and neurogenesis of rat cortical neurons [35]. Here, we found that α-asarone, β-asarone and ATR volatile oil could increase NF68/NF200 transcriptional activities in cultured PC12 cells. In parallel, the potentiating effect of α-asarone, β-asarone and ATR volatile oil in NGF-induced neurofilament expression was fully supported in the co-treatment with low dose of NGF. In addition to neurofilament expression, the ATR-induced neurite outgrowth on PC12 cells was also observed morphologically, which was in line with the role of neurofilaments being the structural components of differentiated neurons. The potentiation of NGF function in the present of ATR volatile oil therefore could be a way for treating neurodegenerative diseases; because the deficiency of NGF in the brain is proposed as one of the causes.

The inhibition of PKA signaling by H89 or KT5720 partially suppressed the ATR volatile oil, α-asarone or β-asarone, induced neuronal differentiation. This partial effect of H89 or KT5720 implies that: (i) asarone or ATR oil penetrates cell membrane and activates gene expression directly; and (ii) other players such as EPAC (exchange proteins directly activated by cAMP), in addition to PKA may also be involved. The aforementioned studies therefore elucidated the involvement of PKA-CREB signaling in α-asarone, β-asarone or volatile oil-induced neuronal differentiation, at least in cultured PC12 cells. ATR volatile oil, α-asarone or β-asarone could induce the pNF68/200-Luc transcriptional activity, neurofilament expression and CREB phosphorylation directly. In parallel, the collaborative effect of asarone or ATR volatile oil with low concentration NGF could boost the effects of NGF in gene expression. Here, we are speculating that ATR volatile oil could be: (i) directly activating intracellular signaling molecules; and (ii) indirectly potentiating NGF-induced signaling by increasing the NGF binding affinity to its Trk A receptor. The second notion is a more favorable one at this stage, and how ATR oil or asarones exerts function on cell membrane receptor should be further investigated.

The combination of α-asarone and β-asarone synergistically increase the neurofilament promoter activity, indicating that the combination of asarone, i.e. ATR volatile oil, might produce synergism to treat neurodegenerative diseases. It was reported that α-asarone and β-asarone were slightly toxic in the acute toxicity test [36]. Reducing the dosages of asarone may reduce the side effects, but the level of pharmacological properties may not be maintained. Here, the combination of two asarone produced maximal activities at low dosages, suggesting the combination might reduce the side effects. This is an example to reveal the co-treatment of toxic compounds could provide higher pharmacological properties with fewer side effects.

## Conclusion

Neurodegenerative diseases could be caused by the deficiency of NGF in the brain. Here, the volatile oil of ATR, a commonly used TCM, is proposed to potentiate the trophic activity of NGF in cultured PC12 cells. The potentiation of ATR volatile oil, α-asarone or β-asarone, in the expression of neurofilaments is mediated by a cAMP-PKA signaling. Therefore, the NGF-potentiating effect of ATR volatile oil, α-asarone or β-asarone, could be considered as a new direction in developing drugs or health food supplements to help the prevention and recovery of neurodegenerative diseases in future.

## Supporting Information

S1 Fig Chemical analysis of volatile oil from ATR. (A) Chemical compositions of volatile oil were analyzed by GC-MS. **(B):** The relative amounts of each chemical were calculated upon peak area of representative chemical. Values were in mean of three individual experiment (*n* = 3). The SEM values were less than 5% of the mean.
(TIF)

S2 Fig Effect of ATR volatile oil, α-asarone or β-asarone, on the viability of PC12 cells. Cultured PC12 cells were treated with the different doses (0.3 to 300 μg/mL) of ATR volatile oil, α-asarone or β-asarone, for 48 hours. Cell viability (using the colorimetric MTT assay) was performed. No significant increase in cell viability was observed. Values are in Mean ± SEM, *n* = 5, each with triplicate samples.
(TIF)

S3 Fig ATR without volatile oil could not induce the transcriptional activation of neurofilament promoters and neurofilament expression in cultured PC12 cells. (A & B) ATR volatile oil, or ATR without volatile oil, was applied onto the cells after transfected with pNF68/200-Luc for 48 hours. The cell lysates were collected to determine the luciferase activity. NGF at 50 ng/ mL served as a control. Values are Means ± SEM, *n* = 3, each with triplicate samples. (C) Cultures were treated with ATR volatile oil, or ATR without volatile oil, at 30 μg/mL for 48 hours. NGF at 50 ng/mL served as a control. The cell lysates were collected to determine the expression of NF68, NF160 and NF200. GAPDH served as loading control, *n* = 4, each with triplicate samples.
(TIF)

## Abbreviations

ATR: Acori Tatarinowii Rhizoma
NGF: nerve growth factor
CREB: cAMP-responsive element binding protein
TCM: traditional Chinese medicine
PKA: protein kinase A
cAMP: cyclic AMP
CNS: central nervous system
