# Supplementary figures and images for: Asarone from Acori Tatarinowii Rhizoma Potentiates the Nerve Growth Factor-Induced Neuronal Differentiation in Cultured PC12 Cells: A Signaling Mediated by Protein Kinase A
Source: PLoS One. 2016 Sep 29;11(9):e0163337. doi: 10.1371/journal.pone.0163337 (PMC5042514; doi:10.1371/journal.pone.0163337)

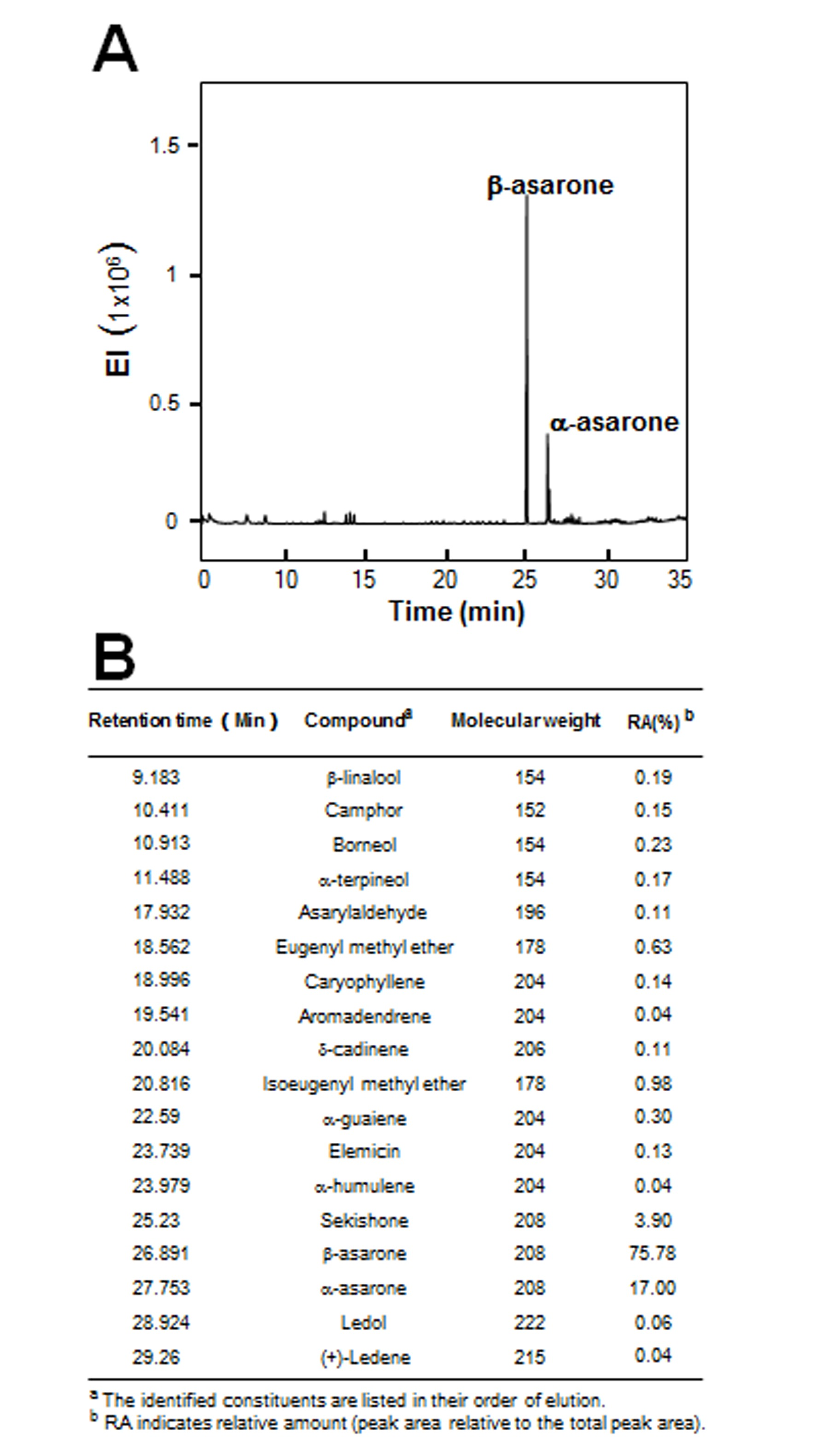

Supplement: S1 Fig — (A) Chemical compositions of volatile oil were analyzed by GC-MS. (B): The relative amounts of each chemical were calculated upon peak area of representative chemical. Values were in mean of three individual experiment (n = 3). The SEM values were less than 5% of the mean. (TIF) [file pone.0163337.s001.tif]

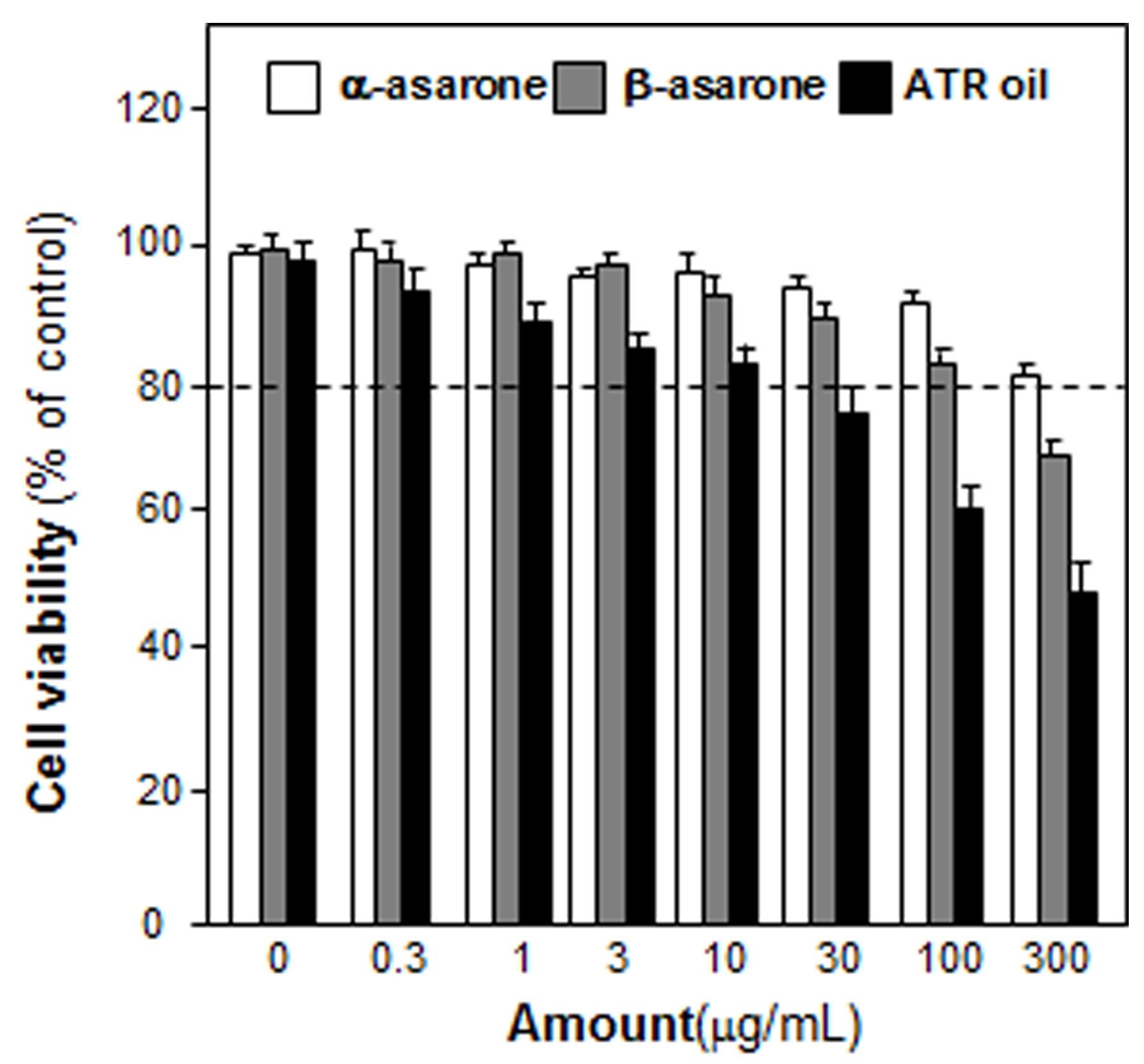

Supplement: S2 Fig — Cultured PC12 cells were treated with the different doses (0.3 to 300 μg/mL) of ATR volatile oil, α-asarone or β-asarone, for 48 hours. Cell viability (using the colorimetric MTT assay) was performed. No significant increase in cell viability was observed. Values are in Mean ± SEM, n = 5, each with triplicate samples. (TIF) [file pone.0163337.s002.tif]

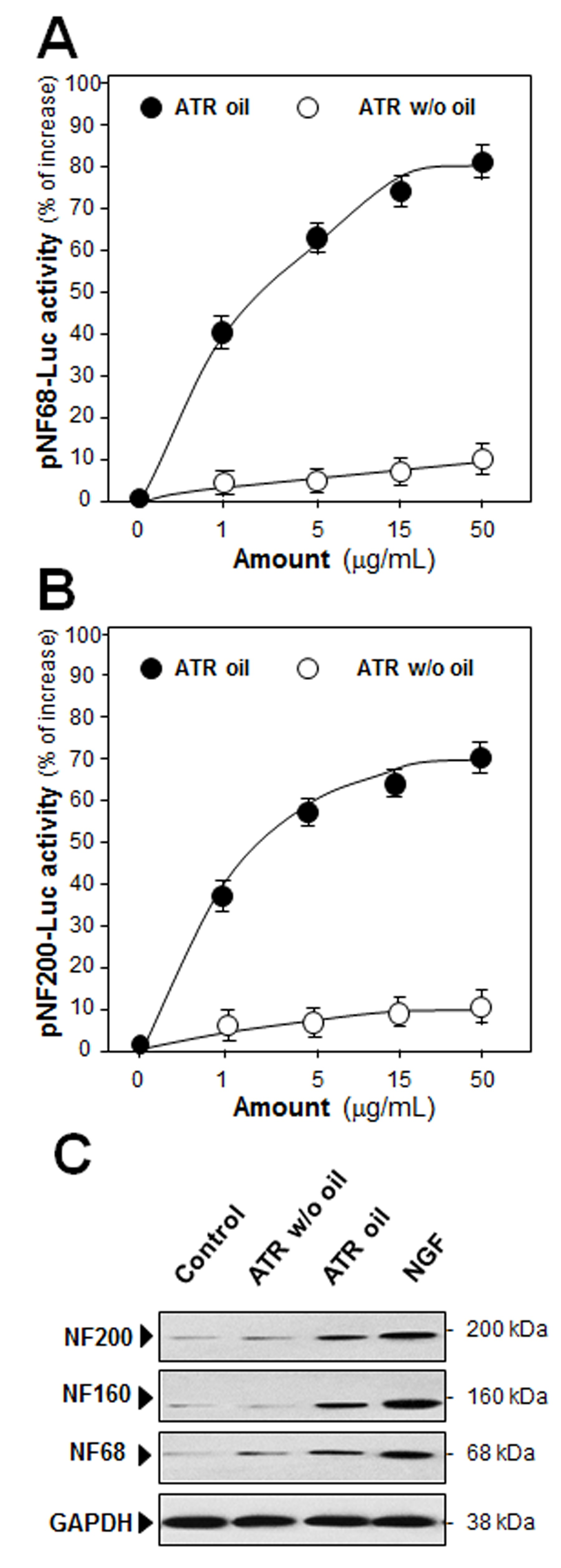

Supplement: S3 Fig — (A & B) ATR volatile oil, or ATR without volatile oil, was applied onto the cells after transfected with pNF68/200-Luc for 48 hours. The cell lysates were collected to determine the luciferase activity. NGF at 50 ng/ mL served as a control. Values are Means ± SEM, n = 3, each with triplicate samples. (C) Cultures were treated with ATR volatile oil, or ATR without volatile oil, at 30 μg/mL for 48 hours. NGF at 50 ng/mL served as a control. The cell lysates were collected to determine the expression of NF68, NF160 and NF200. GAPDH served as loading control, n = 4, each with triplicate samples. (TIF) [file pone.0163337.s003.tif]
